# Impact of Permeable Membrane on the Hydrocyclone Separation Performance for Oily Water Treatment

**DOI:** 10.3390/membranes10110350

**Published:** 2020-11-18

**Authors:** Sirlene A. Nunes, Hortência L. F. Magalhães, Severino R. de Farias Neto, Antonio G. B. Lima, Lucas P. C. Nascimento, Fabiana P. M. Farias, Elisiane S. Lima

**Affiliations:** 1 Department of Fundamental and Social Sciences, Federal University of Paraiba, Areia PB 58397-000, Brazil; sirlene.alves@academico.ufpb.br; 2 Department of Chemical Engineering, Federal University of Campina Grande, Campina Grande PB 58429-900, Brazil; severino.rodrigues@ufcg.edu.br; 3 Department of Mechanical Engineering, Federal University of Campina Grande, Campina Grande PB 58429-900, Brazil; antonio.gilson@ufcg.edu.br (A.G.B.L.); lucaspereira.cn@hotmail.com (L.P.C.N.); limaelisianelima@hotmail.com (E.S.L.); 4 Department of Technology and Development, Federal University of Campina Grande, Sumé PB 58540-000, Brazil; fabianapmf@msn.com

**Keywords:** hydrocyclone, ceramic membrane, multiphase flow, water/oil separation, Ansys software CFX^®^

## Abstract

In the oil industry and academy, the treatment of water contaminated with oil using conventional hydrocyclones and membranes has been an alternative to meet the requirements established by environmental control agencies. However, such equipment is not fully efficient in the treatment of much diluted oily water, with both presenting restrictions in their performance. In this sense, the present work proposes to study the separation process of oily water using a new configuration of hydrocyclone, equipped with a porous ceramic membrane in the conical part’s wall (filtering hydrocyclone). For the theoretical study, a Eulerian–Eulerian approach was applied to solve the mass and momentum conservation equations, and the turbulence model, using the computational fluid dynamics technique. The results of the velocity, pressure and volumetric fraction of the involved phases, and the separation performance of the hydrocyclone, are presented, analyzed, and compared with those obtained with a conventional hydrocyclone. The results confirmed the high potential of the proposed equipment to be used in the separation of the water and oil mixture.

## 1. Introduction

During the productive life of an oil reservoir, it is common to produce water from the reservoir itself, or from the volume of water used in secondary oil recovery, to increase the efficiency of oil recovery. This oily water is commonly known as produced water, and its disposal to the environment is an ongoing concern of the oil and gas sector. Before being discarded or even reused, the produced water must undergo specific and judicious treatment, to meet the standards stipulated by the environmental control agencies worldwide.

For the removal of free oil, the hydrocyclone has proven to be effective equipment in the water/oil separation process. Besides, this equipment has a high processing capacity (requiring little physical space for installation), ease of operation, and low maintenance frequency. These advantages make the hydrocyclone economically viable for this type of activity, and this is quantified by the cost/benefit ratio.

Several studies have been reported in the literature using hydrocyclones in the water/oil separation process [1,2,3,4,5,6,7]. In these studies, the hydrocyclone has been considered as a highly efficient and applicable piecer of equipment, and thus several configurations have been proposed, aiming at its optimization.

Souza et al. [8] and Farias et al. [9] studied ultraviolet oil treatment in a hydrocyclone, using the Eulerian multiphase model. In this study, the authors observed that larger droplet diameter and inlet velocity increase equipment efficiency. Further, Souza et al. [8] found that high temperatures favor the separation process.

Wang et al. [10] presented a three-phase study (gas/liquid/solid) in a hydrocyclone, analyzing the separation process under different dimensions. The authors observed that the geometric aspect directly influences the equipment performance, noting that the long conic section considerably improves the equipment’s efficiency.

Liu et al. [11] studied the water/oil separation process using a magnetic hydrocyclone, reporting that the magnetic field added to the device is capable of increasing the equipment’s separation efficiency by 23.2%.

Al Kayiem et al. [12] investigated the fluid dynamics of a hydrocyclone equipped with both single and double inlets. In this research, the authors observed that double-entry favors the segregation of the phases; moreover, it provides greater separation efficiency (82.3%) when comparing it with the single-entry hydrocyclone (73.7%). Despite its good efficiency in removing free oil from water, the hydrocyclone is not efficient enough to treat oil dispersed in water with oil droplets of small diameters, especially when the oil concentration is low. Under these special conditions, membrane separation processes represent a potential solution to the problem of oily effluent with small-diameter droplets. The main advantages of membrane processes are the retention of oil droplets with dimensions below 10 µm, dispensing with the use of chemical products, and the ability to generate permeate with acceptable quality.

Knowledge of the structure of membranes and their relationship with transport properties are of fundamental importance, especially in terms of improving the understanding of the phenomena involved in the separation problems, providing information that allows the selection of the best structure for a given separation condition. In this sense, ceramic materials generally have characteristics such as chemical inertness, high abrasion resistance, and considerable refractoriness. With this combination of properties, ceramic membranes have been increasingly used for making membranes to be applied in the treatment of oily waters.

Since the first attempts to treat oily membrane effluents in the early 1970s, several studies have been carried out, using different types of membranes [13,14,15,16,17]. One of the main problems reported, associated with the membrane separation processes, is the drop in permeate flow as a result of the accumulation of solute on the membrane surface and impregnation on the permeable surface (fouling).

From the above, taking into account the limitations presented by conventional hydrocyclones and ceramic membranes in the water/oil separation process, when used separately, there is a need to evaluate the use of a new apparatus that simultaneously addresses the hydrocycloning and filtration phenomena. In this sense, some studies using filtering hydrocyclones have been reported in the literature [18,19,20,21] as being applied in the mining industry (solid–liquid separation). However, they are incipient works, and are restricted to some particular hydrocyclone configurations. Furthermore, no works are currently using a filtering cyclonic separator in the water–oil (liquid–liquid) separation process, and studies in this area, therefore, are innovative.

Given the above, this work aims to study the separation process of a mixture of water and oil (produced water) using a new hydrocyclone configuration, using the computational fluid dynamics technique. This device, called a filtering cyclonic separator, has the same working principle as a conventional hydrocyclone, however the conical part is porous (ceramic membrane), and the permeated and concentrated outlets are together at the bottom of the device.

The equipment proposed in this work constitutes an attractive technology, presenting a differentiated geometry capable of significantly reducing the effect of the concentration polarization layer as a result of the swirl flow induced by the tangential entrances of the mixture. Furthermore, the equipment facilitates the additional removal of the permeate flux through the membrane pores and, due to the influence of the formation of the oil core inside the equipment, there is a reduction of oil in the vicinity of the membrane, which prevents the rapid decline in permeate flux in that region, and increases equipment performance.

To evaluate the hydrodynamic flow behavior inside the cyclonic separator and to physically understand the phenomena involved, computational fluid dynamics (CFD) tools were used—more specifically, the Ansys CFX software. Thus, the main causes of the turbulence phenomena and the mechanisms of mass transfer are understood in light of the principles of mass conservation, linear momentum conservation, and mass transport. The expectation is the optimization of the proposed equipment to be applied in situations where the conventional hydrocyclone is not as efficient.

## 2. Methodology

### 2.1. Problem Description

**Figure 1:**
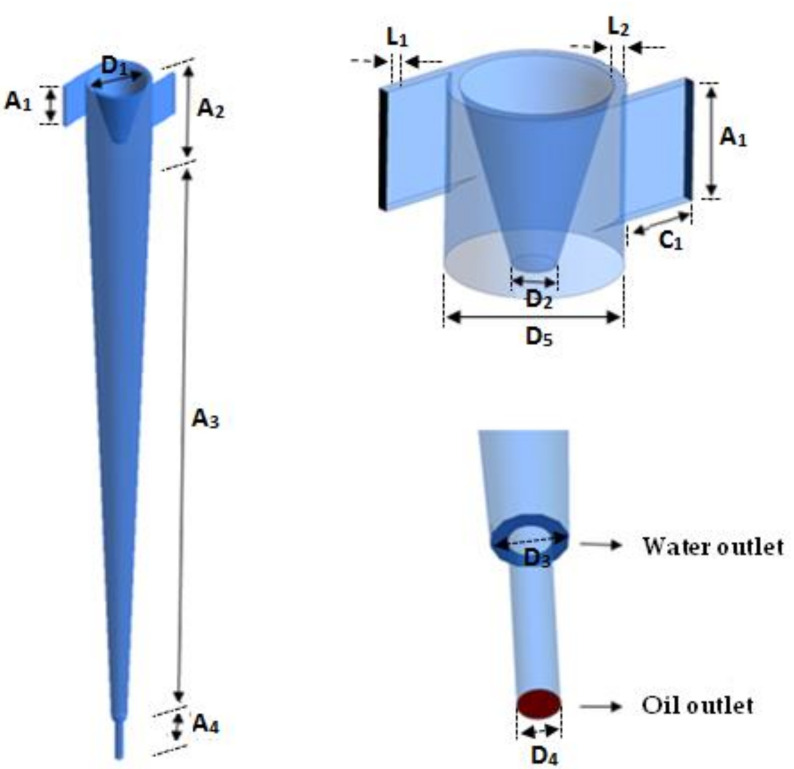
Representation of the cyclonic filter separator.

The study domain corresponds to a filtering cyclonic separator, consisting of a main cone with two tangential inlets, and two axial outlets of different diameters (Figure 1). In the vicinity of the tangential inlets, a tapered trunk was introduced, to direct the flow of oil to one of the axial outlets, and the conical wall is formed by a porous ceramic membrane. To compare the hydrodynamic functioning of the filtering cyclonic separator, a cyclonic separator with the same configuration as the filtering separator was used, but without the porous conical wall. The dimensions of the filtering and traditional cyclonic separators are shown in Table 1.

### 2.2. Computational Domain Generation

To create the domain and generate the cyclonic separator mesh, the Ansys ICEM CFD^®^ software was used. Besides this, to ensure that the mesh leads to coherent numerical results and at the same time requires a lower computational effort, three structured meshes were made with different refinement degrees, aiming at a good distribution of the elements on the study domain. A mesh refining test was carried out, using the mesh convergence index (ICM) method as proposed by [22].

**Figure 2:**
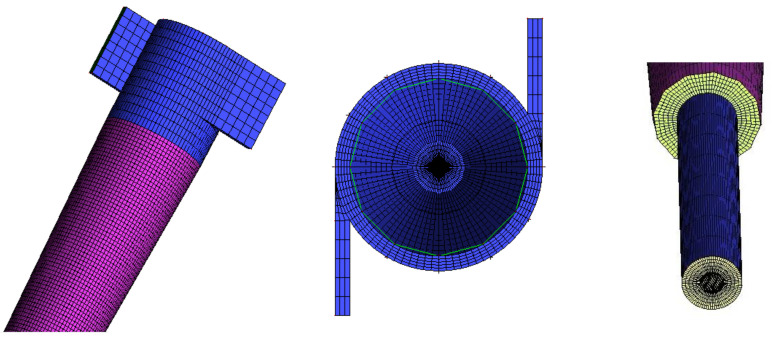
Representation of the numerical mesh of the study domain.

In Figure 2, one of the used meshes, and details of the fluid inlet and outlet region, are represented. In this figure, the good distribution of the elements over the domain can be observed. The generated mesh is refined in the central region, to better capture the velocity gradients close to this region, due to the formation of the internal vortex. The other refinement zone occurs on the walls, to capture the velocity profile and concentration in that region, also influenced by the no-slip condition specified at the wall. The other regions were treated differently to provide smoothness and consistency in the sizes of elements, and to guarantee the quality criteria for the different meshes.

### 2.3. Mathematical Modeling

The mathematical model used to describe the two-phase flow (water/oil) inside the conventional cyclonic separator (without porous membrane) corresponds to a generalization of the mass and linear momentum conservation equations (Navier–Stokes equations), as applied to the Eulerian–Eulerian interfacial transference model [5].

In this model, it is considered that the conservation equations of mass and linear momentum are solved for each of the involved phases (continuous and dispersed), and the coupling between the phases occurs through the interfacial transfer. In addition to these considerations, the following were also adopted:

- Incompressible and Newtonian fluid with constant physical–chemical properties;
- Steady-state and isothermal flow;
- Mass transfer, interfacial momentum, and mass source are disregarded;
- The non-drag interfacial forces (lift forces, wall lubrication, virtual mass, turbulent dispersion and solid pressure) were neglected;
- Constant drag coefficient equal to 0.44, due to the established turbulent flow;
- The geometry walls are static and there is null wall roughness.
- For the filtering hydrocyclone, based on the works of ref. [13,14,15,16], and the considerations already mentioned for the conventional hydrocyclone, the following considerations were made:
- The water stream is a multicomponent mixture of water and oil (solute);
- The composition of the multicomponent water/oil mixture is variable;
- The viscosity and density of the mixture are constant;
- The mass diffusion coefficient of the oil in the water is constant;
- The porous medium (ceramic membrane) has constant permeability and isotropic distribution of it pores;
- The pore obstruction by the solute was neglected (constant porosity);
- The concentration polarization layer is present and its thickness is considered uniform and homogeneous, thus the resistance resulting from the presence of this layer was defined at the fluid–membrane interface (concentration polarization resistance);
- The rate of local permeation is determined by the series resistance theory;
- The non-slip condition on the membrane surface was adopted;
- There is no reaction or adsorption of the solute on the contact surface in the porous medium.

#### 2.3.1. The Governing Equations

For the cyclonic separator the following equations were used.

(a) Mass Conservation Equation:

(1) $$\nabla \cdot \left(f_{\alpha }\rho_{\alpha }{\vec{U}}_{\alpha }\right)=0$$

where the Greek sub-index α represents the phase involved in the two-phase water/oil mixture, and *f*, ρ, and e $\vec{U}$ are the volume fraction, density and velocity vector, respectively.

(b) Momentum Conservation Equation:

(2) $$\nabla \cdot \left[f_{\alpha }\left(\rho_{\alpha }{\vec{U}}_{\alpha }⊗{\vec{U}}_{\alpha }\right)\right]=-f_{\alpha }\nabla p_{\alpha }+\nabla \cdot \left\{f_{\alpha }\mu_{ef}\left[\nabla {\vec{U}}_{\alpha }+{\left(\nabla {\vec{U}}_{\alpha }\right)}^{T}\right]\right\}+{\vec{M}}_{\alpha }$$

where $p_{\alpha }$ is the pressure of phase α, and $M_{\alpha }$ describes the drag force per unit volume on phase *α* due to the interaction with phase *β*, being defined by:

(3) $${\vec{M}}_{\alpha }=\vec{M_{\alpha \beta }^{D}}=C_{\alpha \beta }^{\left(d\right)}\left({\vec{U}}_{\beta }-{\vec{U}}_{\alpha }\right)$$

where $C_{\alpha \beta }^{\left(d\right)}$ corresponds to the dimensionless drag coefficient given by:

(4) $$C_{\alpha \beta }^{\left(d\right)}=\frac{3}{4}\frac{C_{D}}{d_{p}}\;f_{\beta }\rho_{\alpha }\left|{\vec{U}}_{\beta }-{\vec{U}}_{\alpha }\right|$$

where *C_D_* is the drag coefficient and *d_p_* represents the particle diameter. The term $\nabla \cdot \left\{f_{\alpha }\mu_{ef}\left[\nabla {\vec{U}}_{\alpha }+{\left(\nabla {\vec{U}}_{\alpha }\right)}^{T}\right]\right\}$ corresponds to the momentum transfer induced by the interfacial mass transfer, and $\mu_{ef}$ is the effective viscosity, defined by:

(5) $$\mu_{ef}=\mu +\mu_{t}$$

where μ is the dynamic viscosity and $\mu_{t}$ the turbulent viscosity. The turbulent viscosity is a function of turbulent flow intensity and is unknown. It is necessary to use models to predict their value.

In addition to the equations already described, the following mass transport equation was used for the filtering cyclonic separator:

(6) $$\vec{U}\cdot \nabla C=D_{AB}\;\nabla^{2}C$$

where C is the solute concentration and $D_{AB}$ is the mass diffusion coefficient, defined as:

(7) $$D_{AB}=\frac{\mu }{\rho S_{C}}$$

where μ is the dynamic viscosity and $S_{C}$ corresponds to the Schmidt number.

Considering $D_{AB}=4.13\times 10^{-3}T^{1.53}$, with T=20 ℃, the diffusion coefficient used will be $D_{AB}=1.12\times 10^{-8}\;m^{2}/s$.

(c) Turbulence Model:

The turbulence model chosen for the continuous phase was well known for the SST turbulence model. In this model, close to the fluid/membrane interface, the k−ω model is applied and, according to the need, where this model does not show good results, the k−ε model is applied. The choice of the model was made because the cases studied have more pronounced pressure and concentration gradients near the fluid/membrane interface.

(d) Separation Efficiency:

To evaluate the efficiency of water/oil separation, the total efficiency was used, which can be calculated as the ratio between the mass flow rate of oil droplets of a given size $\left(d\right)$ found in the *overflow*, $W_{go}\left(d\right)$, and the mass flow rate of the oil in the feed, $W_{g}\left(d\right)$, given by the equation:

(8) $$G\left(d\right)=100\times \frac{W_{go}\left(d\right)}{W_{g}\left(d\right)}$$

To verify only the amount of oil collected in the *overflow* by the exclusive effect of the hydrocyclone centrifugal field, the reduced separation efficiency $\left(G^{\prime }\right)$ was considered as follows:

(9) $$G^{\prime }=\frac{\left(G-R_{L}\right)}{\left(1-R_{L}\right)}$$

where $R_{L}$ is a parameter that relates the mass flow rate of water collected in the *overflow* $\left(W_{lo}\right)$ and the mass flow rate of water fed in the hydrocyclone $\left(W_{l}\right)$, called the liquid ratio:

(10) $$R_{L}=\frac{W_{lo}\left(d\right)}{W_{l}\left(d\right)}$$

#### 2.3.2. Boundary Conditions

The following boundary conditions were defined at the domain boundaries.

(a) Input:

(11) $$U_{z}=U_{C}$$

(12) $$U_{r}=0$$

(13) $$C=f_{0}$$

where $U_{C}$ is a constant and corresponds to the normal velocity of the input section, $f_{0}$ is the volume fraction of the solute and $U_{r}$ corresponds to the radial velocity calculated from the velocity components $U_{x}$ and $U_{z}$, using the following equation:

(14) $$U_{r}=\;U_{x}.cos\theta +\;U_{z}.sen\;\theta$$

(b) Porous Wall (Permeate):

It was assumed that the permeate flux is equal to the solvent flux at the membrane given by Equation (15). It was also assumed that the non-slip condition for the axial velocity (Equation (16)) was zero, and for the radial velocity (Equation (17)) was equal to the permeation velocity ($U_{r}=U_{w}$), as follows.

(15) $$RU_{r}C=RU_{w}C=D_{AB}\frac{\partial C}{\partial r}$$

(16) $$U_{y}=0$$

(17) $$U_{r}=U_{w}=\frac{\Delta P}{\mu \left(R_{m}+R_{p}\right)}$$

where ΔP is the transmembrane pressure, R is the rejection coefficient of solute by the membrane, $R_{m}$ is the membrane resistance given by Equation (18), and $R_{p}$ is the specific resistance due to the concentration polarization layer, defined by Equation (20).

(18) $$R_{m}=\frac{e}{k_{m}}$$

where e corresponds to the membrane thickness and $k_{m}$ is the membrane permeability [16].

The transmembrane pressure ΔP is defined as the difference between the average pressure of the permeate (${\bar{P}}_{p}$) and the external pressure ($P_{ex}$) to the membrane (atmospheric pressure), given by Equation (19):

(19) $$\Delta P={\bar{P}}_{p}-P_{ex}$$

The specific resistance due to the polarization concentration $R_{p}$ is defined as the change in resistance along the thickness of the polarization layer, given by:

(20) $$R_{p}=\overset{R}{\underset{R-\delta_{p}}{\int }}r_{p}\;d\delta_{p}$$

Considering that the polarization layer has a constant resistance along the thickness, Equation (20) takes the form:

(21) $$R_{p}=r_{p}\delta_{p}$$

where $\delta_{p}$ corresponds to the concentration polarization layer thickness.

The value of $r_{p}$ is calculated using the Kozeny–Carman equation, described by:

(22) $$r_{p}=180\;\frac{{\left(1-\varepsilon_{p}\right)}^{2}}{d_{p}{}^{2}\varepsilon_{p}{}^{3}}$$

where $\varepsilon_{p}$ is the porosity relative to the concentration polarization layer, and $d_{p}$ is the diameter of the solute particles.

The value of the concentration polarization layer thickness, $\delta_{p}$, which measures the distance from the membrane surface to the position where the convective and diffusive fluxes are in equilibrium, and the oil concentration in the layer is close to the inlet concentration, was calculated as follows:

(23) $$\delta_{p}=\frac{1}{0.023}\;D_{h}\times Re^{-0.8}\times Sc^{-1/3}$$

The thickness of the polarized layer is considered constant over the entire membrane, and *Re*, *Sc* and $D_{h}$ are the Reynolds and Schmidt numbers, and the hydraulic diameter, respectively.

The Reynolds number is calculated using Equation (24).

(24) $$R_{e}=\frac{\rho uD_{h}}{\mu }$$

where *u* is the flow velocity, *ρ* is the density, *μ* is the dynamic viscosity and *D_h_* is the hydraulic diameter, given by:

(25) $$D_{h}=D_{C}-D_{TC}$$

where *D_C_* is the cylinder diameter, and *D_TC_* is the diameter of the tapered trunk.

Equations (26) and (27) were used to express the cylindrical part’s area and the velocity.

(26) $$S=\pi r^{2}=\pi {\left(\frac{D_{h}}{2}\right)}^{2}=\frac{\pi }{4}\;\left(D_{C}{}^{2}-D_{TC}{}^{2}\right)$$

(27) $$u=\frac{Q}{S}=\frac{4Q}{\pi \left(D_{C}{}^{2}-D_{TC}{}^{2}\right)}$$

where *Q* is the volumetric flow rate.

Replacing Equations (25), (26) and (27) in Equation (24), we can write:

(28) $$R_{e}=\frac{\rho }{\mu }\;\frac{4Q}{\pi \left(D_{C}{}^{2}-D_{TC}{}^{2}\right)}.\left(D_{C}-D_{TC}\right)$$

so,

(29) $$R_{e}=\frac{4\rho Q}{\pi \mu \;\left(D_{C}+D_{TC}\right)}$$

It is important to note that the volumetric flow rate is given by the sum of the input volumetric flow rate, as follows:

(30) $$Q=Q_{E1}+Q_{E2}$$

where $Q_{E1}=Q_{E2}=Q_{E}$. Thus, we can write:

(31) $$Q=2Q_{E}$$

where *Q_E_* = *u*_E_·*S_E_*, *u*_E_ is the inlet fluid velocity and *S_E_* is the section area of the feed duct.

The Schmidt’s number is given by Equation (32):

(32) $$Sc=\frac{\mu }{\rho }D_{AB}$$

The Linton and Sherwood equation is given by:

(33) $$Sh=\frac{hD_{h}}{D_{AB}}=0.023Re^{0.8}Sc^{1/3}$$

where *h* is the mass transfer coefficient, which can be written by:

(34) $$h=0.023\frac{D_{AB}}{D_{h}}Re^{0.8}Sc^{1/3}$$

(c) Outputs (Concentrated and Diluted):

At the outputs, a pressure of P = 2.1 bar [5], and the conditions given by Equations (35) and (36), were considered:

(35) $$\frac{\partial U_{y}}{\partial y}=0$$

(36) $$\frac{\partial C}{\partial y}=0$$

(d) Non-porous walls:

(37) $$U_{x}=U_{y}=U_{z}=0$$

### 2.4. Studied Cases

The conventional and filtering cyclonic separators were evaluated through numerical simulations using the Ansys CFX^®^ 15.0 software (15, Ansys, Inc., Canonsburg, PA, USA). For the calculations, machines with Intel Core I7-3770 3.40 GHz processor and 16 GB of RAM were used. The simulations were performed using the fixed convergence criterion concerning the residual error–Root Mean Square (RMS) of $10^{-7}\;kg/s$ for the additional and flow variables.

Table 2 shows the parameters’ and materials’ properties adopted in the mathematical model. The solute concentration is inserted into the software as a mass fraction and the interfacial tension of 0.01 N/m was considered.

Table 3 shows the data for the different cases studied. Cases 01 and 03 were used in the study of mesh refining (with and without the porous wall, respectively). Cases 02 and 04, on the other hand, were used to compare the fluid dynamic behavior of the conventional cyclonic separator with that obtained with the filtering cyclonic separator, operating under the same conditions.

## 3. Results and Discussion

### 3.1. Mesh Quality Assessment

The mesh quality analysis was performed using the mesh convergence index method (ICM). For that, three meshes (M1, M2 and M3) were generated with different refinement degrees, using a refining ratio between meshes M1 and M2 of 1.6 and between meshes M2 and M3 of 1.8. These values are within the range proposed by [22]. Table 4 reports the numbers of elements and the simulation times obtained with the different meshes. Based on the previous works reported in the literature [22,23,24] and the mesh refinement study, the mesh M2 was chosen. More details about the mesh refinement study can be found in Nunes [25]. 

**Figure 3:**
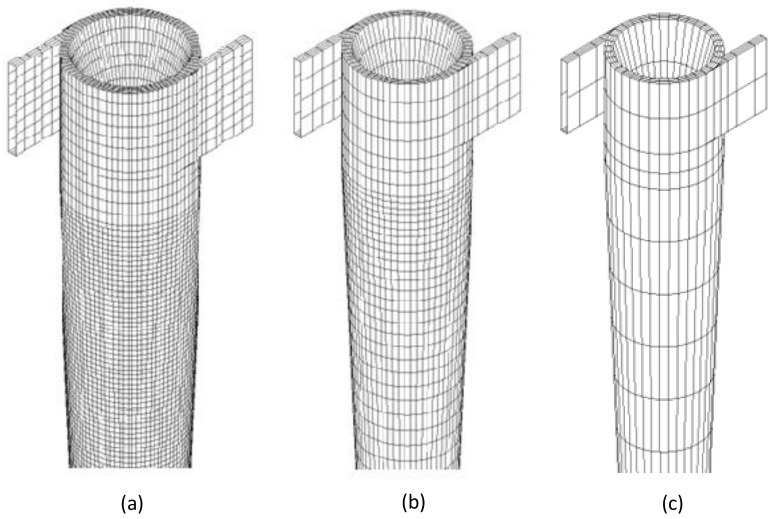
Details of the meshes produced: (**a**) Mesh M1; (**b**) Mesh M2 and (**c**) Mesh M3.

Details of the made meshes are shown in Figure 3. It is important to state that refinement was carried out in the conical region of the study domain, due to the possibility of the presence of high concentration gradients in that region.

### 3.2. Comparative Study between the Conventional Cyclonic Separator and the Cyclonic Filter Separator

**Figure 4:**
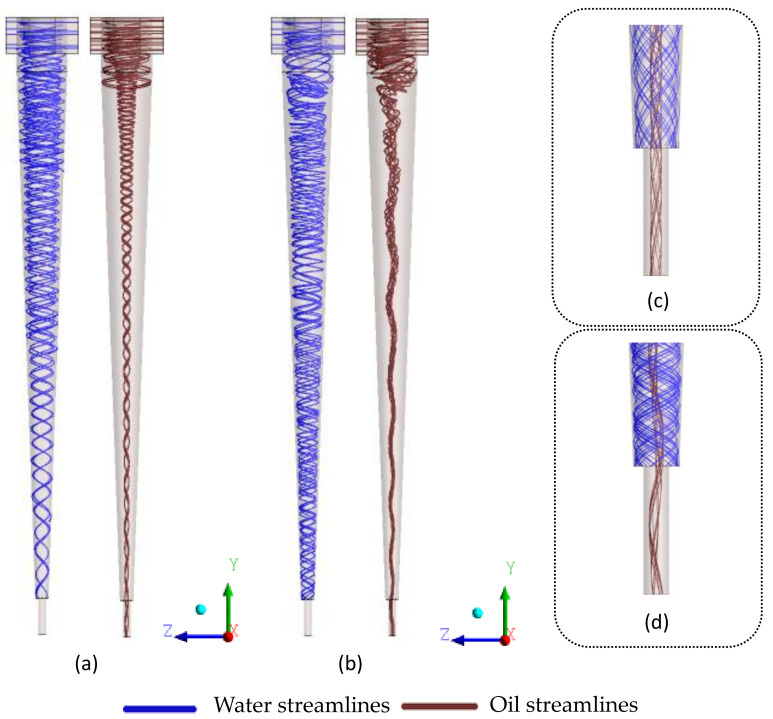
Water and oil streamlines inside the separation equipment. (**a**) Conventional cyclonic separator, (**b**) filtering cyclonic separator, (**c**) detail of the conventional cyclonic separator outlet, and (**d**) detail of the filtering cyclonic separator outlet.

Figure 4 shows the streamlines of the oil and water phases in the filtering and conventional hydrocyclones. An analysis of this figure shows the presence of two distinct fluid streams: a descending spiral shape, close to the wall, and an ascending spiral in the central region. Similar behaviors were found in the literature on conventional hydrocyclones, as in the works reported by [3,5,8,10,26,27,28,29,30]. This behavior was associated with the difference in density, where the spiral flow of the continuous phase (water) tends to flow closer to the separator wall, while the stream of the dispersed phase (oil) flows inside the separator. 

The phase’s behavior is maintained until the end of the process, thus allowing an ideal collection of fluids in the oil and water outlets. However, when comparing the devices formed without or with the porous conical wall (Figure 4a,b) it can be observed that the oil stream in the center of the separator shows an unstable behavior of the oil core when compared to the cyclonic separator, without the porous conical wall. This fact can be explained by the presence of the permeate flux perpendicular to the ceramic membrane. However, it can be seen with the help of Figure 4c,d that the behavior of the collection of fluids in the outlet region of the cyclonic separators is not altered.

**Figure 5:**
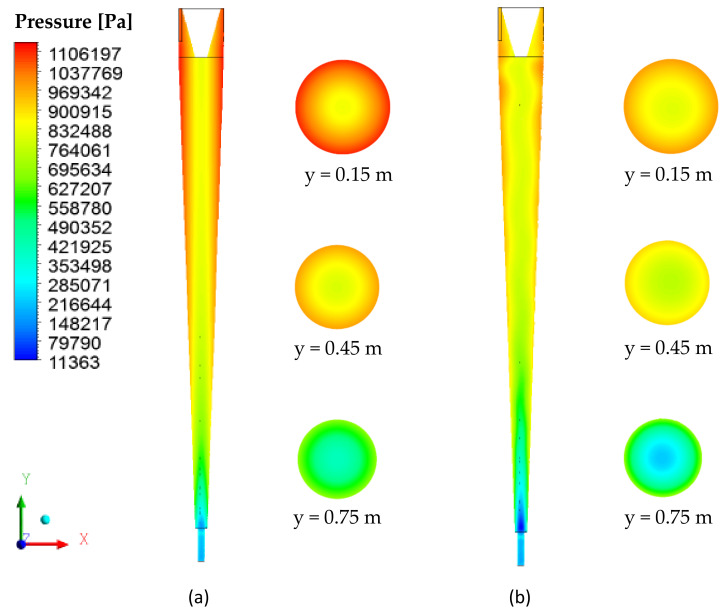
Pressure field on the xy and xz planes. (**a**) Conventional cyclonic separator and (**b**) filtering cyclonic separator.

Figure 5 illustrates the pressure distributions along the xy and xz longitudinal planes. Note that the pressure decreases radially towards the center from the separator wall, reaching its lowest value close to the outlets. A dimilar behavior was observed in ref. [3,4,8] when studying traditional hydrocyclones, and in ref. [5] with a geometry similar to that of the present research, considering the impermeable conical wall. This behavior is associated with the centrifugal force.

**Figure 6:**
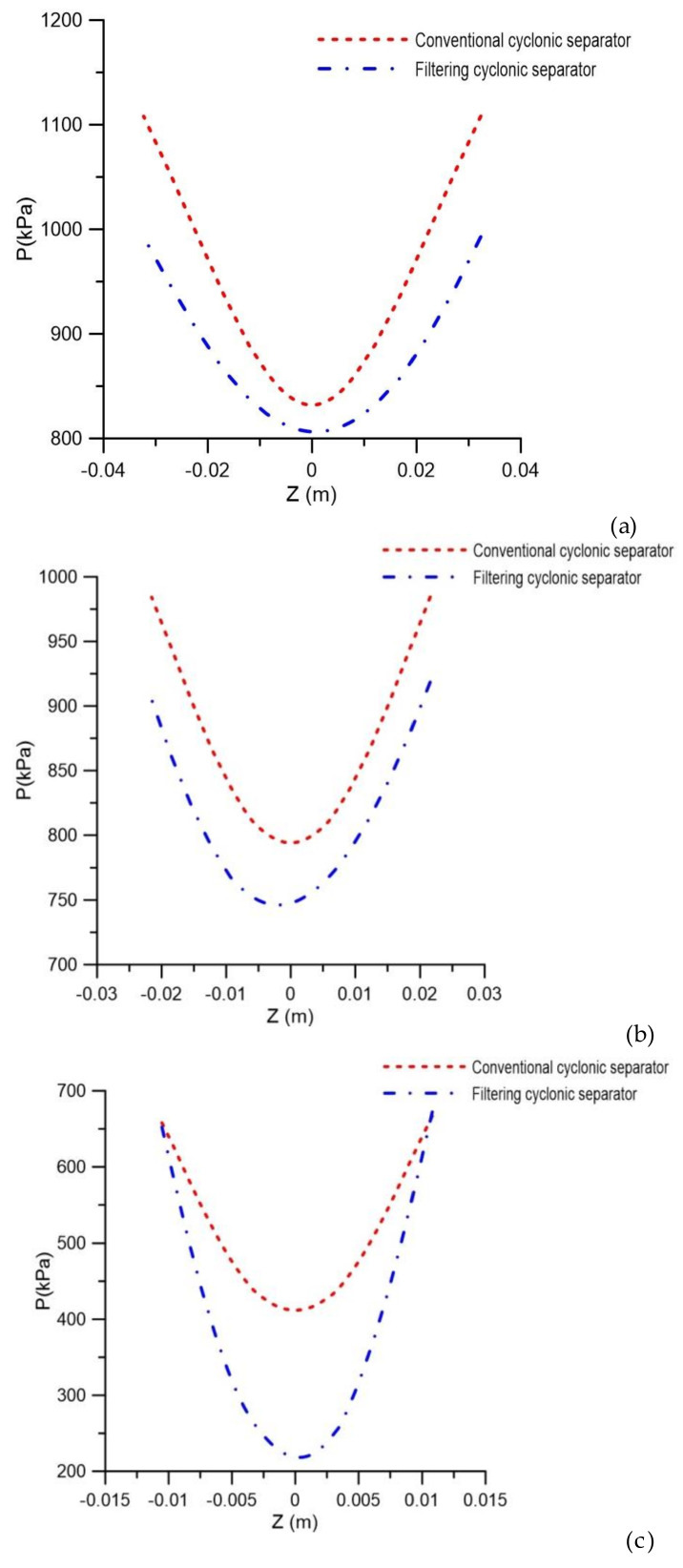
Pressure profile in conventional and filtering hydrocyclones at positions (**a**) y = 0.15 m, (**b**) y = 0.45 m and (**c**) y = 0.75 m.

When comparing the cyclonic separators without and with the ceramic membrane (Figure 5a,b, respectively), it was observed that, with the same feeding speed, the pressure gradient in the vicinity of the cylindrical and conical walls was less intense for the filtering cyclonic separator than it was for the equipment without the ceramic membrane (conventional cyclonic separator). Similar behavior was also obtained by [31] when studying a filtering hydrocyclone in the solid particle/water separation process. The lower intensity of the pressure gradient in the filtering separator is because the pores of the membrane represent an additional outlet of liquid, previously not available during the operation of the conventional cyclonic separator (where water necessarily had to escape through the underflow holes or overflow). This fact can also be seen in Figure 6, which presents the pressure profiles in the axial positions y = 0.15 m, y = 0.45 m and y = 0.75 m along the separators.

**Figure 7:**
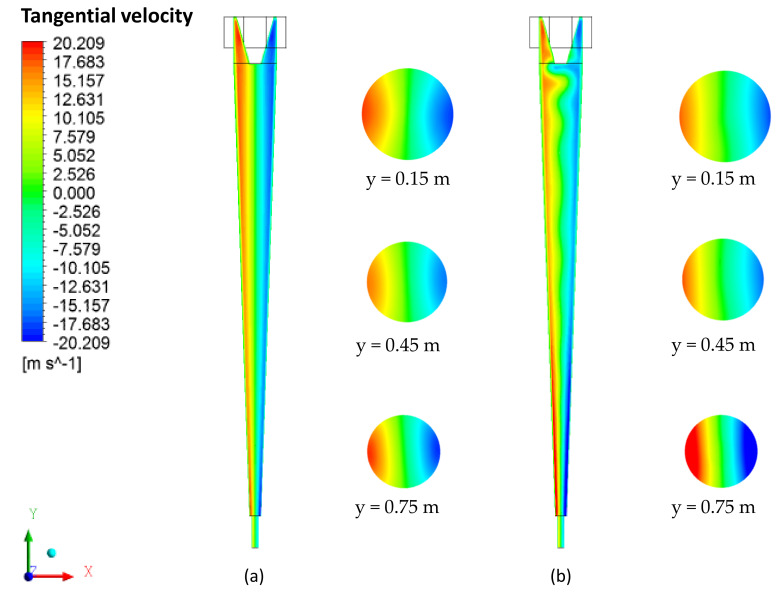
Water tangential velocity field in the yz plane. (**a**) Conventional hydrocyclone and (**b**) filtering hydrocyclone.

**Figure 8:**
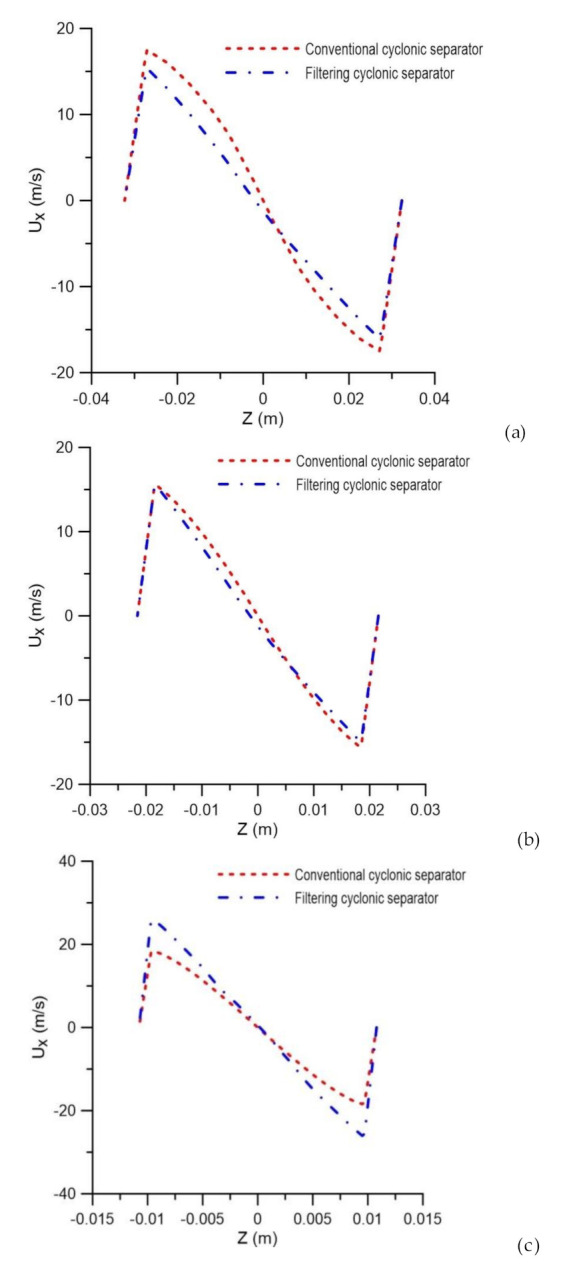
Tangential velocity profile in conventional and filtering hydrocyclones at the positions (**a**) y = 0.15 m, (**b**) y = 0.45 m and (**c**) y = 0.75 m.

The tangential velocity field on the yz plane, passing through the central axis of the cyclonic separator, is shown in Figure 7. It appears that the tangential velocity increases in intensity radially in the direction of the central axis (zero velocity) for the cylindrical and conical walls of the evaluated devices, reaching maximum values in the vicinity of the cyclonic separator walls. A similar behavior is reported in [4,5,11,32,33,34]. However, when comparing the separators without and with the ceramic membrane, the influence of the permeate flux through the ceramic membrane on the hydrodynamic behavior of the components of tangential velocities is perceived. This behavior is confirmed in Figure 8, which describes the tangential velocity profiles.

**Figure 9:**
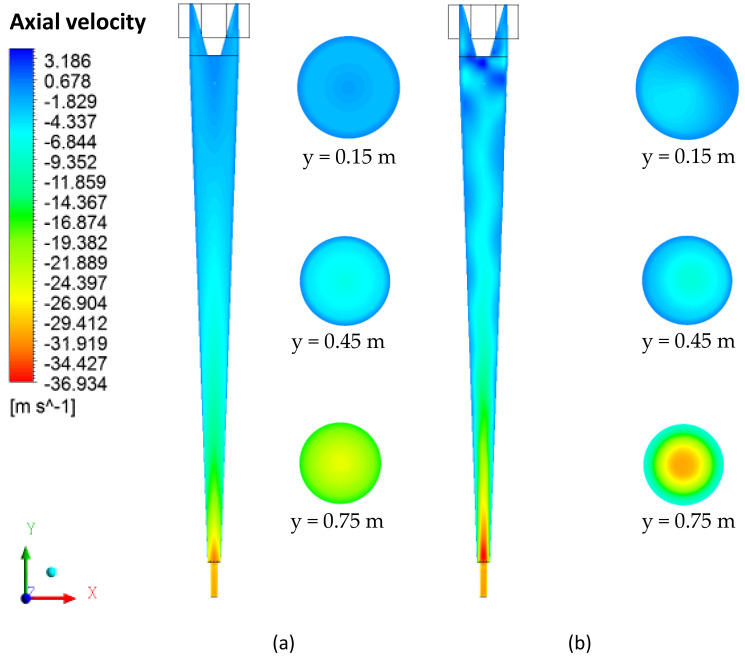
Water axial velocity field in the yz plane. (**a**) Conventional hydrocyclone and (**b**) filtering hydrocyclone.

The axial velocity field on the yz plane, passing through the central axis of the cyclonic separator, is shown in Figure 9. In general, it is observed that the axial velocity components increase as they approach the oil and water outlets, and have the highest intensities in the vicinity of the separator axis. Similar behavior was observed in [5] when similarly evaluating a device. However, the presence of the ceramic membrane leads to the disorderly character of the behavior, as compared to the cyclonic separator with an impermeable wall (conventional cyclonic separator). 

**Figure 10:**
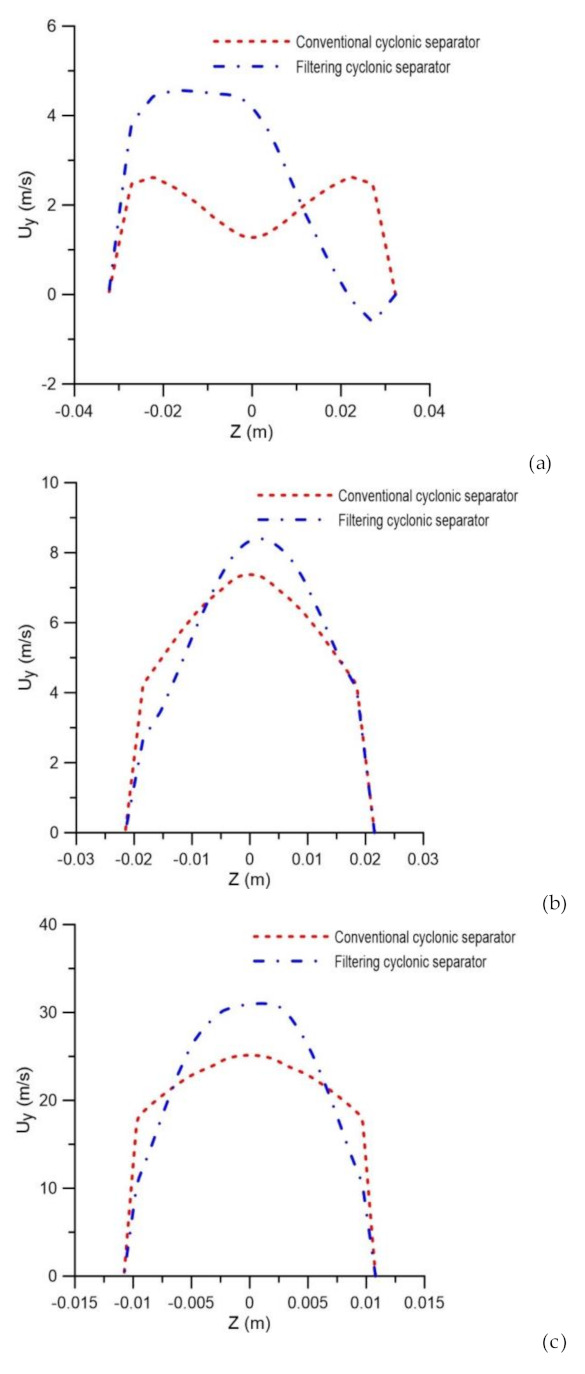
Axial velocity profile in the conventional and filtering hydrocyclones at the positions (**a**) y = 0.15 m, (**b**) y = 0.45 m and (**c**) y = 0.75 m.

It is possible to observe in Figure 10, which describes the axial velocity profile at positions y = 0.15 m, y = 0.45 m and y = 0.75 m, that the filtration phenomena modified the axial velocity profiles in the cyclonic separator.

It appears that the filtering cyclonic separator has higher axial speeds in the central region of the equipment when compared with the cyclonic separator with an impermeable wall. It can also be observed that the highest values obtained for axial speed are located at the end of the equipment (close to the exits). This is because, in that region, the axial linear momentum prevails over the angular momentum, thus reducing the intensity of the turbulence, especially in the vicinity of the oil and water outlets. This behavior was also observed by [4,35].

**Figure 11:**
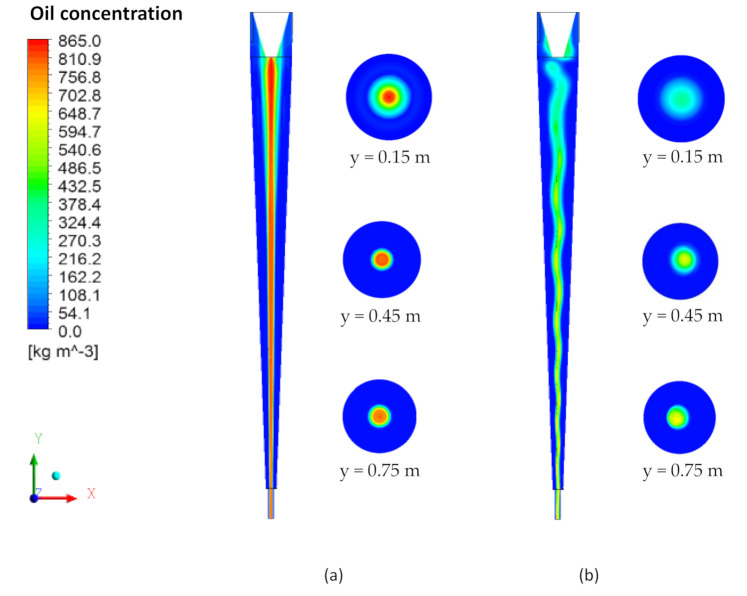
Oil concentration field in the yx and xz planes at positions y = 0.15 m, y = 0.45 m and y = 0.75 m. (**a**) Conventional cyclonic separator and (**b**) filtering cyclonic separator.

Figure 11 illustrates the oil concentration field on the xy and xz planes passing through the central axis of the conventional and filtering cyclonic separators at different transversal positions. It is possible to observe that the oil tends to be located in the center of the separator from the beginning of the flow in the two devices, as already observed in the streamlines. However, the behavior of the oil in the central region of the filtering cyclonic separator changes, forming a more diluted and undulating oil stream. This fact can be explained by the change in the fluid dynamic behavior of the fluids due to the presence of the permeate flux perpendicular to the ceramic membrane, as well as the increase in the axial speed of the water observed in Figure 9 and Figure 10.

**Figure 12:**
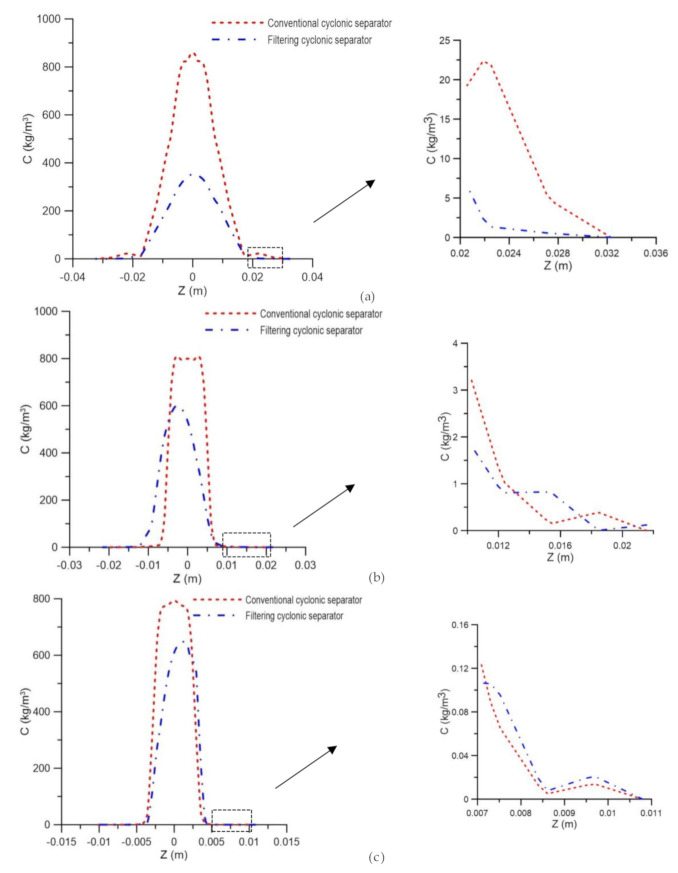
Oil concentration profile in conventional and filtering hydrocyclones at the positions (**a**) y = 0.15 m, (**b**) y = 0.45 m and (**c**) y = 0.75 m.

The oil concentration profiles represented in Figure 12 confirm that the oil concentration is lower in the cyclonic separator with a ceramic membrane. This fact is due to the migration of water in the direction of the conical wall of the equipment, which causes the oil stream to disperse and suffer a greater effect from the mixture of fluids. Figure 12 also shows the oil concentration profiles for the cyclonic separators with and without the ceramic membrane close to the conical wall (region highlighted in Figure 12), calculated in the axial positions of 0.15 m, 0.45 m and 0.75 m. It is possible to observe, in Figure 12a, a greater concentration of oil in the cyclonic separator without a ceramic membrane (conventional hydrocyclone), as had already been observed along the axial position. However, the different behaviors of the oil concentration profiles in the vicinity of the conical wall are observed in Figure 12c, in the region close to the outlets, as a result of the greater flux of water allowed by the membrane pores in this region, which causes an increase in the oil concentration for the equipment with an impermeable wall (filtering cyclonic separator), a fact that interferes in the fluid separation efficiency, which will be discussed in detail later.

Table 5 shows the inlet and outlet mass flow rate for each fluid in the conventional and filtering cyclonic separators. It is possible to observe that the water mass flow rate at the axial outlet of the filtering cyclonic separator decreased when compared to the conventional cyclonic separator. This fact occurs due to the permeate flux leaving the membrane and due to the increase in the oil concentration close to the concentrate outlet. We state that the permeate flux (0.74 kg/s, almost 10.71% of the feed flow rate) corresponds to clean water since the membrane rejection index is unified. This is very important from the social point of view, and shows clearly the impact of this research on the world. Table 6 shows the total separation efficiency, liquid ratio and reduced separation efficiency of the conventional cyclonic separator and the filtering cyclonic separator. It is possible to observe that the filtration associated with the separation process was able to modify the liquid ratio, which was increased concerning the conventional cyclonic separator.

It is believed that the migration of the suspension stream in the direction of the wall caused interference in the relationships between the volumes of the free and forced vortexes, which, due to the turbulence generated, collaborated in the modification of the liquid fraction that was directed to each of the exits. It should also be noted that the calculation of the reduced separation efficiency in the filtering cyclonic separator does not take into account the permeate flux, which, due to the additional flow, minimizes the reduced separation efficiency. A similar fact was observed in [1,36], when studying numerically and experimentally the optimization of the separation processes in filtering hydrocyclones.

Under the same operational conditions, the total efficiency and reduced efficiency of the filtering cyclonic separator suffered a decrease of approximately 5%, when compared to the cyclonic separator without the presence of the porous medium (conventional hydrocyclone). This difference is attributed to the way these parameters are calculated. It is possible to observe, through the tangential velocity profiles, that the fluid has been dampened in the rotational movement inside the separator. As the fluid’s spiral movement decreases, the centrifugal force inside the equipment is reduced, which leads to the least amount of particles collected. This leads to an increase in the oil concentration close to the membrane, which can induce the formation of the polarization concentration layer.

From the petroleum industry point of view, oil production has increased considerably throughout the world over the years. As a result, the search for new types/processes of produced water treatment (that is associated with that of oil production) has become crucial. These new alternatives take into account factors as diverse as the forms of oil in water (free, emulsified, and/or dissolved), the destination of treated water (disposal, injection and/or reuse), the location of the production equipment/facilities, the legislative, technical and financial feasibility of the process and equipment involved, as well as the availability of the infrastructure.

In the case of international legislation (present day) for the disposal of production water at sea, the maximum limits for total oils and greases vary from 15 mg·L^−1^ to 50 mg·L^−1^, depending on the country. In Brazil, the permitted value is 29 mg·L^−1^ (simple monthly arithmetic mean), with a maximum daily value of 42 mg·L^−1^ [37]. To comply with environmental legislation, the oil industry has used certain equipment, such as air floats, hydrocyclones (offshore installations), bed coalescers, and gravitational separators (onshore installations). Despite being used today, these processes have some disadvantages, such as long residence time, the use of high-cost special chemicals, the generation of solid waste, and their low efficiencies, especially when the oil drops have diameters in the order of micrometers, and tensioactive agents are present, which are very common in emulsions.

For more severe conditions (small oil droplets and emulsified oil), the membrane separation process has been used. As advantages of this technique, we can mention the retention of oil drops with dimensions smaller than 10 µm, the low operating cost when compared to usual processes, the rendering as unnecessary the use of chemicals, and the ability to generate permeates with acceptable quality (complying with current environmental legislation). Despite these advantages, during operation, there is a rapid decline in the permeate flow, which is mainly attributed to the concentration polarization and fouling phenomena.

Concentration polarization consists of the formation of a concentration gradient in the fluid layer immediately adjacent to the membrane surface (concentration boundary layer). Fouling, on the other hand, is related to the blocking of the membrane pores by oil drops and other contaminants present in the produced water, and the accumulation of particles on the membrane surface (deposition, precipitation and adsorption), which cause an increase in operating pressure (transmembrane pressure), a reduction in the facility’s efficiency, and a reduction in the membrane life. In industry, the concentration polarization effect is controlled by increasing the speed of the mixture in the feed (increased fluid turbulence) or air bubbling. Fouling, on the other hand, is controlled by pre-treating the feed stream, changing the operating conditions, cleaning the membranes (chemical and physical), and even modifying the membranes.

Given the above, it can be said that the application of a filtering hydrocyclone presents itself as a very robust alternative to replace or even operate in conjunction with existing traditional systems. The main advantages of the proposed equipment are related to those of conventional hydrocyclones and membranes; for example, the low operating and maintenance cost, the fact that it does not require the use of chemicals as inputs, as well as the high quality of the permeate, which complies with current legislation. Despite this, new studies are needed to better understand the process and equipment, which will make it possible to expand/optimize their application in the treatment of water polluted by oil and/or other contaminants. The main challenges are related to operational problems, mainly those resulting from the phenomena of concentration polarization and fouling in the membrane, and the geometric and thermo-fluid dynamic of the hydrocyclone, which strongly affect the separation efficiency.

## 4. Conclusions

Based on the numerical results obtained in the water/oil separation process via a cyclonic separator, it can be concluded that the mathematical model used successfully described the three-dimensional behavior of the multiphase and multicomponent flow within the conventional and filtering cyclonic separators. Besides this, this study proved that a porous conical wall in the equipment causes instability in the central oil core, due to the presence of a permeate flow perpendicular to the ceramic membrane, and that the filtration associated with the hydrocycloning process is capable of altering the performance of the cyclonic separator. Both conventional and filtering cyclonic separators tend to concentrate the oil in the central region throughout the flow; however, for high oil concentrations, the core expands and the oil particles approach the porous wall of the filtering hydrocyclone. The pressure and oil concentration inside the conventional hydrocyclone are higher than those in the filtering hydrocyclone, mainly close to the wall and entrance region. Finally, it was observed that, under fixed operational conditions, both the total and reduced efficiencies of the filtering cyclonic separator suffered reductions of approximately 5% when compared to the conventional cyclonic separator, due to the method of calculating these parameters.

## Figures and Tables

**Table 1:** Dimensional parameters of filtering and conventional cyclonic separators.

Tangential Inlets (mm)	Height (A_1_)	50
	Length (C_1_)	50
	Width (L_1_)	5
Upper Conical Part (mm)	Height (A_2_)	75
	Width (L_2_)	5
	Top Diameter (D_1_)	65
	Bottom Diameter (D_2_)	18
Cylindrical Section (mm)	Height (A_2_)	75
	Diameter (D_5_)	70
Conical Section (mm)	Height (A_3_)	725
Annular Outlet (mm)	Diameter (D_3_)	18
Tubular Outlet (mm)	Diameter (D_4_)	10
	Height (A_4_)	50

**Table 2:** Thermal, physical, chemical and geometrical parameters of the porous wall and mixture fluids (T = 293.15 K).

**Membrane**	Permeability	$1.39\times 10^{-15}\;m^{2}$ [16]
	Polarization layer thickness	0.255 mm [16]
	Porosity	0.4
**Water**	Density	$997\;kg/m^{3}$
	Viscosity	$8.889\times 10^{-4}\;Pa\cdot s$
	Molar mass	18.05 kg/kmol
**Oil**	Density	$868.7\;kg/m^{3}$
	Viscosity	0.985 Pa·s
	Molar mass	873 kg/kmol
	The average oil drop diameter	0.1 mm

**Table 3:** Operational parameters used in the simulations.

Case	Input Velocity (m/s)	Oil Volumetric Fraction (%)	Membrane Rejection Index R (-)
01	5	5.0	-
02	15	7.5	-
03	5	5.0	1
04	15	7.5	1

**Table 4:** Mesh information created for the convergence index analysis.

Mesh	Number of Elements	Simulation Time	
		Cyclonic Separator	Filtering Separator
M1	337.360	1 d 4 h 17′26″	3 d 8 h 4′2″
M2	71,352	3 h 10′44’’	21 h 38′40″
M3	10,571	23′22″	17′4″

**Table 5:** The mass flow rate of fluids at the inlets and outlets of the hydrocyclones.

Separator			Mass Flow Rate (kg/s)					
	Water	Oil		Water			Oil	
	Input	Input		Annular Output	Tubular Outlet	Membrane	Annular Output	Tubular Outlet
Conventional Cyclonic	6.91	0.48		5.19	1.72	-	2.02 × 10^−4^	0.48
Filtering Cyclonic	6.91	0.48		4.41	1.76	0.74	1.99 × 10^−4^	0.46

**Table 6:** The separation efficiency of oil and the liquid ratio of the hydrocyclones.

Separator	Total Efficiency (%)	Liquid Ratio (%)	Reduced Efficiency (%)
Conventional Cyclonic	99.95	24.94	99.94
Filtering Cyclonic	96.07	25.46	94.72
